# Perception of Smile Aesthetics of Patients with Anterior Malocclusions and Lips Influence: A Comparison of Dental Professionals', Dental Students,' and Laypersons' Opinions

**DOI:** 10.1155/2020/8870270

**Published:** 2020-10-14

**Authors:** Khaled Khalaf, Zahra Seraj, Hesham Hussein

**Affiliations:** Department of Preventive and Restorative Dentistry, University of Sharjah, P.O. Box 27272, Sharjah, UAE

## Abstract

**Objectives:**

The aim of the study was to evaluate the perception of smile aesthetics in patients with varying degrees of anterior crowding and spacing among dental specialists, dentists, dental students, and laypersons and to assess the effect of the lips position.

**Materials and Methods:**

Clinical photos of cases of anterior crowding (mild, moderate, and severe) and spacing (mild, moderate, and severe) with and without the associated lips were used. The images were evaluated by 250 persons including dental specialists, dentists, dental students, and laypersons using the rating scale method, (extremely unaesthetic to extremely aesthetic, 1 to 5, respectively). Nonparametric tests were used to analyse the data on SPSS version 24.

**Results:**

According to the 250 responses, the aesthetic perception of all severities of anterior crowding and mild and moderate spacing was found to be more aesthetic when displayed with the lips (*p* < 0.05). Additionally, the higher the level of dental education, the lower the aesthetic perception with and without the lips (*p* < 0.05).

**Conclusions:**

The lips play a significant role in improving the aesthetics of crowded anterior teeth and spaced anterior teeth. Dental educational level influences the aesthetic perception of anterior crowding and spacing. This may have an impact on treatment planning and need for orthodontic intervention.

## 1. Introduction

The number of patients seeking orthodontic treatment due to aesthetic concern is on the rise [1]. While this may be influenced by social media in young adults, according to Langlois et al. [2], physical attractiveness receives more positive judgements and may impact academic performance in both children and adults. Furthermore, Kiyak [3] reported that patients seek orthodontic treatment not to improve oral function but to enhance their appearance and increase their chances of social acceptance. Therefore, the impact of malocclusion on aesthetics can positively or negatively affect the quality of life, interpersonal relationships, and psychological well-being [4, 5].

It has been reported that smile plays a crucial role in facial aesthetics [6]. It ranks second as the most important facial feature for attractiveness with the eyes ranking first [7]. The lips define the aesthetic zone and form the outer frame of the smile and can reveal or conceal tooth and tissue defects and asymmetries [8, 9]. It could be said that the greater the visual awareness of teeth during smiling, the greater the impact of anterior malocclusions on the psychosocial aspects of people's self-perceived facial aesthetics. Hence, an attractive smile is one of the most important treatment objectives along with creating a functional occlusion.

Anterior malocclusions are often the first malocclusions spotted as they lie in the frontal area of the mouth and are usually noticed in the early mixed dentition. In the sagittal plane, anterior malocclusions mainly include increased overjet and anterior crossbite. Vertically, anterior malocclusions include deep overbite (the upper incisors overlap the lower incisors by >1/3^rd^ of their height) and open bite. Transverse anterior malocclusions include a midline displacement and a crossbite. Anterior malocclusions affecting the perimeters of the dental arches include crowding and spacing. Crowding frequently occurs in the anterior segments of the dental arches, while spacing is less common [10]. Different classifications of crowding and spacing have been reported, but according to the most common ones, mild crowding is defined as a crowding of 0–3 mm, moderate as 4–8 mm, and severe as >8 mm [11]. On the other hand, mild spacing is defined as spacing of 2–3 mm, moderate as 4–8 mm, and severe as >8 mm [12].

It was found that gender and education level influence the perception of aesthetic smile [13]. In addition, laypersons and dental professionals would view the aesthetic appearance of a set of teeth differently depending on the view given to them (dental view/facial view) [13]. Even within the dental field, specialists may have different perceptions of the aesthetic smile to general dentists, and general dentists may have different perceptions to dental students. Furthermore, orthodontists tend to be more critical of occlusion than general dentists, other specialists, and laypersons. They would also rate specific characteristics such as arch form, spacing, and crowding as more or less aesthetic [14, 15]. Dental asymmetries such as gingival margins, tooth wear, and dental midline shift are also perceived differently between different dental professionals, where orthodontists are more critical than the rest [16].

Although there have been many studies that investigated facial soft tissue characteristics, such as diagnostic similarities between soft-tissue analysis performed on cephalometric radiographs, profile photographs, and a skeletal cephalometric analysis [17], the relationships between subjective evaluations of smiles judged from clinical photographs and objective evaluations were measured from the smile mesh program [18] and compared the milled with prototyped mock-ups when designing smile characteristics [19]. There is a lack of consensus regarding the impact of different degrees of anterior crowding and spacing on the perception of smile aesthetics among dental professionals and laypersons. This is more so when the position of the lips is considered as a potentially contributing factor to masking anterior crowding and spacing.

Thus, the aim of this study was to evaluate the perception of smile aesthetics in patients with varying degrees of crowding and spacing among laypersons, dental students, dentists, and dental specialists and to assess whether the position of the lips influence this perception. The results of the study yield invaluable information helping us identify whether there is a difference in the perception of aesthetics between individuals with different levels of dental education and how orthodontic and/or restorative treatment indications may change accordingly. They would also enable us to identify whether the lips influence this perception by masking certain malalignments, which makes this study the first of its kind.

## 2. Materials and Methods

### 2.1. Participants

Ethical approval was sought and subsequently granted by the Research Ethics Committee at the University of Sharjah (reference number: REC-18-02-12-05-S). The participants of this questionnaire-based study were 250 adults (86 males and 164 females; mean age ± SD: 25.9 ± 9.58 years) who were divided into six groups according to their level of dental education as follows: I: 25 dental specialists, II: 25 dentists, III: 25 fifth year dental students, IV: 25 fourth year dental students, V: 25 third year dental students, and VI: 125 laypersons. The students were all enrolled in the undergraduate Bachelor of Dental Surgery (BDS) programme at the University of Sharjah, Sharjah, United Arab Emirates. The specialists included faculty members in the same university as well as from different private clinics/health centres within the same country. Laypersons included members of the public who had no dental education background and were randomly selected from the United Arab Emirates general public. Both genders were included in each subgroup of the participants. Specialists should have finished their specialisations at least 2 years prior to be included in the study, and laypersons should have completed a college education but no dental background. Specialists included licenced orthodontists, prosthodontists, oral and maxillofacial surgeons, endodontists, and paediatric dentists. All specialists completed a formal 3-year postgraduate training programme leading to the award of certificate of completion of specialist training and the placement on the specialist list of the dental register.

### 2.2. Data Collection

**Figure 1:**
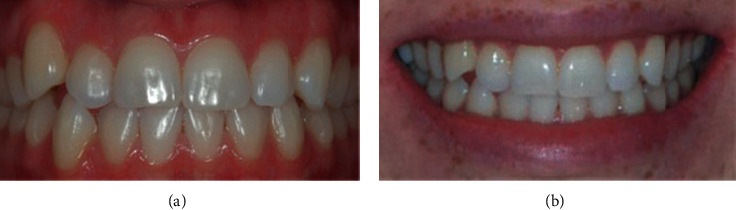
Mild crowding.

**Figure 2:**
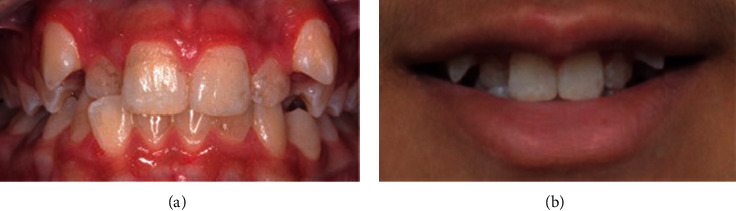
Moderate crowding.

**Figure 3:**
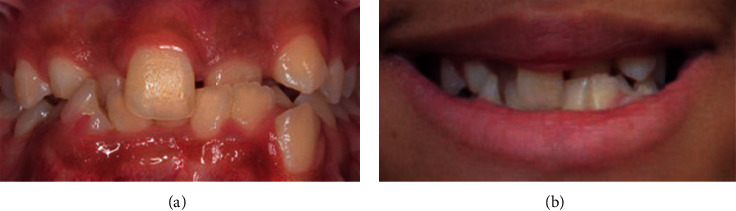
Severe crowding.

**Figure 4:**
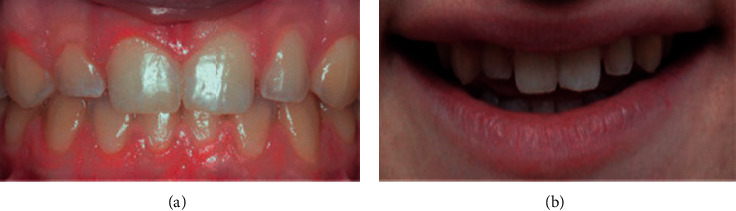
Mild spacing.

**Figure 5:**
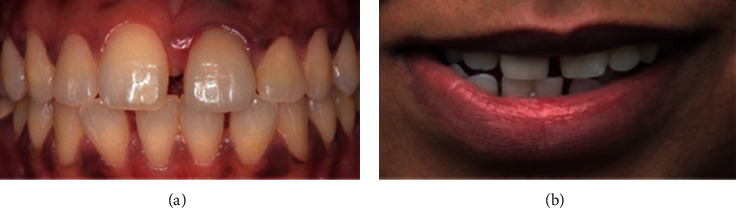
Moderate spacing.

**Figure 6:**
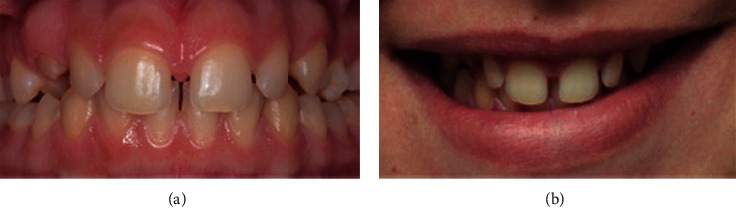
Severe spacing.

Data were collected by a specialist orthodontist (KK) and two general dentists (ZS and HA) between December 2018 and February 2019. The participants' opinions regarding their perception of smile aesthetics of anterior malocclusions and lips position were recorded anonymously using a digital questionnaire hosted on Google Forms. All participants were recruited on a voluntary basis in the university campus as well as areas of the public. The questionnaire included twelve frontal photos of anterior crowding and spacing of varying degrees of severity (mild, moderate, and severe crowding and mild, moderate, and severe spacing), with and without the lips visible. Participants were asked to rate each photograph on a 5-point Likert scale ranging from 1, extremely unaesthetic to 5, extremely aesthetic. Figures 1–6 show the clinical photographs used in our study. The questionnaire was completed by 366 persons, of whom 250 were selected after screening the responses for inaccuracies by the author who was blinded to the participants' variables such as dental education and gender. The final section of the questionnaire collected demographic data regarding the participants' age and gender, in addition to history of direct experiences of orthodontic treatment.

### 2.3. Statistical Analyses

Statistical analyses were performed using SPSS® ver. 24.0. Nonparametric tests, Wilcoxon signed-rank test and Kruskal–Wallis test, were applied to analyse the data. Additionally, a post hoc analysis was conducted using Mann–Whitney U test. Statistical significance was considered at *p* < 0.05. Statistical advice was sought from a formally qualified statistician prior to choosing and conducting the analyses that were carried out by one of the authors.

## 3. Results

Table 1 shows the differences in the perception of attractiveness of anterior crowding and spacing with and without the lips. All cases with the exception of severe spacing had a significantly higher aesthetic rating (*p* < 0.05) with the lips when compared to those without the lips.

The data analysis for demographic characteristics of the subjects included in the study is presented in Table 2. According to the 250 responses, the higher the level of dental education, the lower the aesthetic perception with and without the presence of lips. No significant differences were detected in the perception of aesthetics among genders, except for moderate crowding without the lips (*p* < 0.05). Older age groups had a significantly increased perception of aesthetics for cases with mild spacing but without the lips (*p* < 0.05).

The comparison of the perception of attractiveness of anterior crowding with and without the lips among the six groups of raters ((a) in Table 3) revealed significant differences (*p* < 0.05) for cases of mild crowding without ((a) in Table 3) and with ((b) in Table 3) the lips and moderate ((d) in Table 3) and severe ((f) in Table 3) crowding with the lips. In these cases, generally, dental specialists had a low aesthetic perception of cases with anterior crowding compared with dentists, dental students, and laypersons.

The comparison of the perception of attractiveness of anterior spacing with and without the lips among the six groups of raters ((a) in Table 4) revealed significant differences (*p* < 0.05) for cases of mild spacing with the lips ((b) in Table 4) and moderate ((c) in Table 4) and severe ((e) in Table 4) spacing without the lips. In these cases, generally, dental specialists had a low aesthetic perception of cases with anterior spacing compared with dentists, dental students, and laypersons.

## 4. Discussion

In social psychology, the “what is beautiful is good” stereotype was tested by Dion et al. in 1972 [20], and it denotes that human beings attribute positive qualities to attractive individuals and vice versa. Additionally, according to the “halo-effect” [21–23], physical attractiveness and interpersonal qualities are systematically linked. In a meta-analysis conducted by Langlois et al [2], physical attractiveness received more positive judgement and was noted to be an advantage for both children and adults in terms of academic performance. Furthermore, children with normal dento-facial appearance were reported to be more beautiful, desirable as friends, and intelligent than those with Angle class II or III [24]. These societal expectations start early in childhood and last a lifetime [25]. Therefore, it may be said that attractiveness plays a rather important role in our daily lives, and it may have an impact on various aspects of our lives such as occupation, marriage, and fulfilment [4, 5].

The purpose of this study was to evaluate the perception of smile aesthetics in cases with varying degrees of anterior crowding and spacing among dental specialists, dentists, dental students, and laypersons and to assess the effect of the presence of lips on this perception. The lips define the aesthetic zone and form the outer frame of the smile [8]. They may reveal or conceal tooth and tissue defects and asymmetries [9], and according to the findings of our study, the lips significantly altered the perception of aesthetics for all cases of anterior malocclusions (*p* < 0.05) except those with severe spacing (*p* > 0.05). Therefore, the morphology of the lips should be considered during the treatment planning process of patients with anterior malocclusions due to their influence on the aesthetics of smile as found by the current study and others [26].

The findings of our study suggest that most categories of anterior crowding (except moderate crowding with the lips and severe crowding without the lips) and mild and moderate spacing with the lips were rated more aesthetically by laypersons than specialists (*p* < 0.05). It is not possible to compare the findings of our study with previous investigations due to the lack of similar previous investigations. However, previous investigations assessing the impact of buccal corridors on smile attractiveness have reported findings contrary to the findings of our study, whereby they found no significant differences in the perception of smile aesthetics between dental professionals and laypersons [27, 28]. The aesthetic perception of a smile is subjective and may be influenced by a number of factors including media, socioeconomic status, and cultural background [13]. Regardless, dental professionals appear to be more critical in evaluating smile aesthetics as compared to patients, which may be due to their scientific background regarding the principles of smile design and proportions [29, 30]. Furthermore, differences in the perception of smile aesthetics arose at an interprofessional level, whereby dental specialists rated the photographs depicting mild crowing with the lips significantly less aesthetic than all the other groups (*p* < 0.05). The findings of this study are in agreement with previous studies [31, 32], although none of the previous studies have investigated different degrees of anterior crowing and spacing. Therefore, this emphasizes the need for orthodontists to assess patient's perception of the aesthetics of their anterior malocclusion, especially those with mild crowding, prior to providing orthodontic treatment and to be aware not to impose his/her aesthetic ideals on the patient as a history of dental training may act as a bias in the decision-making process.

According to the literature, poor dental aesthetics has been associated with decreased self-confidence and are considered to be a social, occupational, and academic disadvantage [25]. Younger generations are attaching increasing importance to all aspects of their appearance [33]. However, according to the present study, no difference in the perception of smile aesthetics was detected between ages, except for mild spacing without the lips, whereby older individuals perceived the malocclusion to be more aesthetic than younger individuals (*p* < 0.05). This shows that younger age groups may be more critical than older age groups to some features of anterior malocclusion, a finding which has also been reported in other studies that investigated the impact of age on smile perception of gingival display and the presence of a black triangle between the maxillary central incisors [34].

There are some conflicting findings regarding gender and its effect on the perception of dental aesthetics, whereby some studies have reported female patients to be more concerned and critical with their dental appearance than male patients [35–38]. However, these findings were not confirmed in other studies [39, 40], and according to the findings of the current study, only moderate crowding without the lips was rated to be less aesthetic by female persons (*p* < 0.05).

Considering this, it is worthy to mention that the female-male sample ratio was not equal in this study and is a limitation that should be addressed in the future to further confirm these findings. Other limitations include lack of a comparison photo with no anterior malocclusion and the use of nondigitally modelled photos with a standardized lip shape, tooth shade, and shape and gingival display. Furthermore, it would be interesting in future studies to investigate the lips characteristics, i.e., size and shape as well as gingival display on smile aesthetics of patients with various features of anterior malocclusion.

## 5. Conclusion

1. Dental specialists tended to have a low aesthetic perception of cases with anterior crowding and spacing compared with dentists, dental students, and laypersons.
2. The lips play a significant role in improving the aesthetics of anterior crowding of teeth.
3. Clinicians should bear in mind the differences in perception of smile aesthetics in the management of patients with anterior crowding and spacing.

## Figures and Tables

**Table 1:** Differences in the perception of attractiveness of anterior crowding and spacing with and without the lips.

Case	Mean ± SD	*p* value
Mild crowding		
Without lips	3.228 ± 0.835	0.0001
With lips	3.976 ± 0.850	

Moderate crowding		
Without lips	1.256 ± 0.670	0.0001
With lips	2.096 ± 0.826	

Severe crowding		
Without lips	1.148 ± 0.687	0.0001
With lips	1.296 ± 0.728	

Mild spacing		
Without lips	2.912 ± 0.787	0.002
With lips	3.092 ± 0.835	

Moderate spacing		
Without lips	1.956 ± 0.844	0.0001
With lips	2.436 ± 0.868	

Severe spacing		
Without lips	1.924 ± 0.891	0.494
With lips	1.972 ± 0.833	

**Table 2:** Differences in the perception of attractiveness of anterior crowding and spacing with and without upper lips position and according to demographic characteristics and groups.

Case	Demographics			Groups
	Gender	Age	History of orthodontic treatment	
Mild crowding				
Without lips	0.480	0.493	0.961	0.002
With lips	0.195	0.371	0.231	0.002

Moderate crowding				
Without lips	0.037	0.102	0.152	0.796
With lips	0.708	0.897	0.232	0.015

Severe crowding				
Without lips	0.485	0.316	0.940	0.070
With lips	0.753	0.612	0.657	0.001

Mild spacing				
Without lips	0.211	0.045	0.817	0.583
With lips	0.611	0.323	0.048	0.009

Moderate spacing				
Without lips	0.428	0.059	0.635	0.069
With lips	0.490	0.164	0.962	0.006

Severe spacing				
Without lips	0.865	0.091	0.798	0.004
With lips	0.205	0.154	0.437	0.616

**Table 3:** Differences among groups in the perception of attractiveness of anterior crowding with and without the lips.

Group	I	II	III	IV	V	VI
(a) Mild crowding without the lips							
I						
II	0.434					
III	0.381	0.460				
IV	0.392	0.617	0.912			
V	0.032	0.550	0.141	0.239		
VI	0.001	0.120	0.009	0.027	0.381	

(b) Mild crowding with the lips						
I						
II	0.004					
III	0.001	0.166				
IV	0.026	0.317	0.044			
V	0.012	0.782	0.397	0.340		
VI	0.0001	0.171	0.819	0.039	0.446	

(c) Moderate crowding without the lips						
I						
II	0.533					
III	0.542	0.944				
IV	0.918	0.419	0.445			
V	0.918	0.419	0.445	1.000		
VI	0.409	0.945	0.885	0.308	0.308	

(d) Moderate crowding with the lips						
I						
II	0.574					
III	0.535	0.844				
IV	0.888	0.421	0.396			
V	0.242	0.534	0.756	0.133		
VI	0.016	0.057	0.156	0.004	0.209	

(e) Severe crowding without the lips						
I						
II	0.077					
III	0.077	1.000				
IV	0.077	1.000	1.000			
V	0.077	1.000	1.000	1.000		
VI	0.660	0.125	0.125	0.125	0.125	

(f) Severe crowding with the lips						
I						
II	0.723					
III	0.525	0.293				
IV	0.934	0.655	0.540			
V	0.934	0.615	0.556	0.967		
VI	0.037	0.062	0.006	0.025	0.018	

**Table 4:** Differences among groups in the perception of attractiveness of anterior spacing with and without the lips.

Group	I	II	III	IV	V	VI
(a) Mild spacing without the lips						
I						
II	0.554					
III	0.554	0.137				
IV	0.786	0.652	0.408			
V	0.554	0.137	1.000	0.408		
VI	0.517	0.910	0.192	0.775	0.192	

(b) Mild spacing with the lips						
I						
II	0.958					
III	0.656	0.671				
IV	0.575	0.533	0.282			
V	0.891	0.915	0.705	0.441		
VI	0.042	0.031	0.005	0.184	0.013	

(c) Moderate spacing without the lips						
I						
II	0.958					
III	0.656	0.671				
IV	0.575	0.533	0.282			
V	0.891	0.915	0.705	0.441		
VI	0.042	0.031	0.005	0.184	0.013	
VI	0.532	0.203	0.032	0.119	0.098	

(d) Moderate spacing with the lips						
I						
II	0.068					
III	0.477	0.226				
IV	0.421	0.327	0.874			
V	0.685	0.16	0.783	0.689		
VI	0.006	0.274	0.023	0.043	0.017	

(e) Severe spacing without the lips						
I						
II	0.671					
III	0.087	0.241				
IV	0.022	0.103	0.610			
V	0.002	0.012	0.139	0.312		
VI	0.656	0.896	0.108	0.028	0.001	

(f) Severe spacing with the lips						
I						
II	0.914					
III	0.914	1.000				
IV	0.345	0.387	0.387			
V	0.231	9.192	0.192	0.859		
VI	0.903	0.943	0.943	0.285	0.131	

## Data Availability

All relevant data are included in the manuscript. Any additional data can be provided by the corresponding author upon request.

## References

[1] Theobald A. H., Wong B. K. J., Quick A. N., Thomson W. M. (2006). The impact of the popular media on cosmetic dentistry. *The New Zealand Dental Journal*.

[2] Langlois J. H., Kalakanis L., Rubenstein A. J., Larson A., Hallam M., Smoot M. (2000). Maxims or myths of beauty? A meta-analytic and theoretical review. *Psychological Bulletin*.

[3] Kiyak H. A. (2008). Does orthodontic treatment affect patients’ quality of life?. *Journal of Dental Education*.

[4] Broder H. L., Slade G., Caine R., Reisine S. (2000). Perceived impact of oral health conditions among minority adolescents. *Journal of Public Health Dentistry*.

[5] de Paula D. F., Santos N. C. M., da Silva É. T., Nunes M. F., Leles C. R. (2009). Psychosocial impact of dental esthetics on quality of life in adolescents. *The Angle Orthodontist*.

[6] Miller F. D., Kalin R. S., Meyer P. A. (1970). The effects of temporal variables on the acquisition of human avoidance behavior. *Psychonomic Science*.

[7] Goldstein R. E. (1969). Study of need for esthetics in dentistry. *The Journal of Prosthetic Dentistry*.

[8] Garber D. A., Salama M. A. (2000). The aesthetic smile: diagnosis and treatment. *Journal of Periodontol*.

[9] Davis N. C. (2007). Smile design. *Dental Clinics of North America*.

[10] Yu X., Zhang H., Sun L., Pan J., Liu Y., Chen L. (2019). Prevalence of malocclusion and occlusal traits in the early mixed dentition in Shanghai, China. *Peer Journal*.

[11] Naish H., Dunbar C., Crouch-Baker J. (2016). Does a true knowledge of dental crowding affect orthodontic treatment decisions?. *The European Journal of Orthodontics*.

[12] Shakeel Q. K., Babur A., Adeel Q. K., Hassan M. (2014). Prevalence of malocclusion and its relation with crowding and spacing. *Pakistan Oral & Dental Journal*.

[13] Flores-Mir C., Silva E., Barriga M. I., Lagravère M. O., Major P. W. (2004). Lay person’s perception of smile aesthetics in dental and facial views. *Journal of Orthodontics*.

[14] Kokich V. O., Kokich V. G., Kiyak H. A. (2006). Perceptions of dental professionals and laypersons to altered dental esthetics: asymmetric and symmetric situations. *American Journal of Orthodontics and Dentofacial Orthopedics*.

[15] Roden-Johnson D., Gallerano R., English J. (2005). The effects of buccal corridor spaces and arch form on smile esthetics. *American Journal of Orthodontics and Dentofacial Orthopedics*.

[16] Pinho S., Ciriaco C., Faber J., Lenza M. A. (2007). Impact of dental asymmetries on the perception of smile esthetics. *American Journal of Orthodontics and Dentofacial Orthopedics*.

[17] Nucera R., Lo Giudice A., Bellocchio M., Spinuzza P., Caprioglio A., Cordasco G. (2017). Diagnostic concordance between skeletal cephalometrics, radiograph-based soft-tissue cephalometrics, and photograph-based soft-tissue cephalometrics. *European Journal of Orthodontics*.

[18] Schabel B. J., McNamara J. A., Franchi L., Baccetti T. (2009). Q-sort assessment vs visual analog scale in the evaluation of smile esthetics. *American Journal of Orthodontics and Dentofacial Orthopedics*.

[19] Lo Giudice A., Ortensi L., Farronato M., Lucchese A., Lo Castro E., Isola G. (2020). The step further smile virtual planning: milled versus prototyped mock-ups for the evaluation of the designed smile characteristics. *BMC Oral Health*.

[20] Dion K., Berscheid E., Walster E. (1972). What is beautiful is good. *Journal of Personality and Social Psychology*.

[21] Andreoni J., Petrie R. (2008). Beauty, gender and stereotypes: evidence from laboratory experiments. *Journal of Economic Psychology*.

[22] Callan M. J., Powell N. G., Ellard J. H. (2007). The consequences of victim physical attractiveness on reactions to injustice: the role of observers’ belief in a just world. *Social Justice Research*.

[23] Smith S. M., McIntosh W. D., Bazzini D. G. (1999). Are the beautiful good in Hollywood? An investigation of the beauty-and-goodness stereotype on film. *Basic and Applied Social Psychology*.

[24] Shaw W. C. (1981). The influence of children’s dentofacial appearance on their social attractiveness as judged by peers and lay adults. *American Journal of Orthodontics*.

[25] Sarver D. M., Ackerman M. B. (2003). Dynamic smile visualization and quantification: part 2. Smile analysis and treatment strategies. *American Journal of Orthodontics and Dentofacial Orthopedics*.

[26] Scott C. R., Goonewardene M. S., Murray K. (2006). Influence of lips on the perception of malocclusion. *American Journal of Orthodontics and Dentofacial Orthopedics*.

[27] Martin A. J., Buschang P. H., Boley J. C., Taylor R. W., McKinney T. W. (2007). The impact of buccal corridors on smile attractiveness. *The European Journal of Orthodontics*.

[28] Ritter D. E., Gandini L. G., Pinto A. D. S., Locks A. (2006). Esthetic influence of negative space in the buccal corridor during smiling. *The Angle Orthodontist*.

[29] Bhuvaneswaran M. (2010). Principles of smile design. *Journal of Conservative Dentistry*.

[30] Levin E. I. (1978). Dental esthetics and the golden proportion. *The Journal of Prosthetic Dentistry*.

[31] Machado A. W., McComb R. W., Moon W., Gandini L. G. (2013). Influence of the vertical position of maxillary central incisors on the perception of smile esthetics among orthodontists and laypersons. *Journal of Esthetic and Restorative Dentistry*.

[32] Albino J. E., Tedesco L. A., Conny D. J. (1984). Patient perceptions of dental-facial esthetics: shared concerns in orthodontics and prosthodontics. *The Journal of Prosthetic Dentistry*.

[33] Tüzgiray Y. B., Kaya B. (2013). Factors affecting smile esthetics. *Turkish Journal of Orthodontics*.

[34] Sriphadungporn C., Chamnannidiadha N. (2017). Perception of smile esthetics by laypeople of different ages. *Progress in Orthodontics*.

[35] Tin-Oo M. M., Saddki N., Hassan N. (2011). Factors influencing patient satisfaction with dental appearance and treatments they desire to improve aesthetics. *BMC Oral Health*.

[36] Samorodnitzky-Naveh G. R., Geiger S. B., Levin L. (2007). Patients’ satisfaction with dental esthetics. *The Journal of the American Dental Association*.

[37] Liepa A., Urtane I., Richmond S., Dunstan F. (2003). Orthodontic treatment need in Latvia. *The European Journal of Orthodontics*.

[38] Ong E., Brown R. A., Richmond S. (2006). Peer assessment of dental attractiveness. *American Journal of Orthodontics and Dentofacial Orthopedics*.

[39] Springer N. C., Chang C., Fields H. W. (2011). Smile esthetics from the layperson’s perspective. *American Journal of Orthodontics and Dentofacial Orthopedics*.

[40] Hamdan A. M., Al-Omari I. K., Al-Bitar Z. B. (2007). Ranking dental aesthetics and thresholds of treatment need: a comparison between patients, parents, and dentists. *The European Journal of Orthodontics*.

